# A Genetic Model of Impulsivity, Vulnerability to Drug Abuse and Schizophrenia-Relevant Symptoms With Translational Potential: The Roman High- vs. Low-Avoidance Rats

**DOI:** 10.3389/fnbeh.2019.00145

**Published:** 2019-07-05

**Authors:** Osvaldo Giorgi, Maria G. Corda, Alberto Fernández-Teruel

**Affiliations:** ^1^Department of Life and Environmental Sciences, University of Cagliari, Cagliari, Italy; ^2^Medical Psychology Unit, Department of Psychiatry and Forensic Medicine, School of Medicine, Institute of Neurosciences, Universitat Autónoma de Barcelona, Barcelona, Spain

**Keywords:** Roman high- and low-avoidance rats, genetic animal model, impulsivity, schizophrenia-relevant symptoms, sensation/novelty seeking, vulnerability to drug abuse, mesoaccumbens and mesocortical dopaminergic pathways

## Abstract

The bidirectional selective breeding of Roman high- (RHA) and low-avoidance (RLA) rats for respectively rapid vs. poor acquisition of active avoidant behavior has generated two lines/strains that differ markedly in terms of emotional reactivity, with RHA rats being less fearful than their RLA counterparts. Many other behavioral traits have been segregated along the selection procedure; thus, compared with their RLA counterparts, RHA rats behave as proactive copers in the face of aversive conditions, display a robust sensation/novelty seeking (SNS) profile, and show high impulsivity and an innate preference for natural and drug rewards. Impulsivity is a multifaceted behavioral trait and is generally defined as a tendency to express actions that are poorly conceived, premature, highly risky or inappropriate to the situation, that frequently lead to unpleasant consequences. High levels of impulsivity are associated with several neuropsychiatric conditions including attention-deficit hyperactivity disorder, obsessive-compulsive disorder, schizophrenia, and drug addiction. Herein, we review the behavioral and neurochemical differences between RHA and RLA rats and survey evidence that RHA rats represent a valid genetic model, with face, construct, and predictive validity, to investigate the neural underpinnings of behavioral disinhibition, novelty seeking, impulsivity, vulnerability to drug addiction as well as deficits in attentional processes, cognitive impairments and other schizophrenia-relevant traits.

## Introduction

Impulsivity is a multifaceted trait that involves premature responses or actions without foresight, often leading to adverse or maladaptive consequences. Impaired impulse control and deficits in attention are characteristic, and in some cases core aspects, of several neuropsychiatric conditions, such as attention-deficit hyperactivity disorder, obsessive-compulsive disorder, antisocial behavior, mania, schizophrenia, and drug addiction (Dalley et al., 2011; Jupp and Dalley, 2014; Hoptman, 2015; Hayward et al., 2016; Chase et al., 2017; Dalley and Robbins, 2017).

Research on the pathophysiological substrate of impulsivity has benefited from the availability of both, psychometrical and experimental measures of this multifaceted construct combined with functional magnetic resonance imaging (fMRI) studies in humans, and valid models to assess impulsive behavior either as a trait or a state in animals.

An example of animal model of impulsivity is represented by the Roman high- (RHA) and low-avoidance (RLA) lines/strains of rats. These rats are bidirectionally selected and bred for their rapid (RHA) vs. extremely poor (RLA) ability to acquire the two-way active avoidance response in a shuttle box. More than four decades of research have characterized RHA rats as less fearful, anxious and stress-sensitive than their RLA counterparts (Steimer and Driscoll, 2003; Driscoll et al., 2009; Río-Álamos et al., 2017a,b). Remarkably, compared with RLAs, RHA rats display an impulsive phenotype, as shown by their response profiles in the delayed response latency 20 (DRL-20), schedule-induced polydipsia, and 5-choice serial reaction time (5CSRT) tasks (Zeier et al., 1978; Moreno et al., 2010; Merchán et al., 2019; see “The Roman Rats: A Genetic Model of Differential Anxiety, Novelty Seeking, Impulsivity, Attention Deficits and Associated Traits” section). Moreover, relative to RLAs, RHA rats display several impairments in schizophrenia-relevant phenotypes, such as prepulse inhibition (PPI; Oliveras et al., 2015; Río-Álamos et al., 2019), latent inhibition (LI; Fernández-Teruel et al., 2006), spatial working memory (Oliveras et al., 2015) and reversal learning (Río-Álamos et al., 2019) as well as a trend towards a reduction of some types of social behavior (Coppens et al., 2012; Del Rio et al., 2014; see “RHA Rats as a Model of Deficits in Attentional Processes, Cognitive Impairments and Other Features Relevant for Schizophrenia Research” section). These behavioral profiles are consistent with findings of a reduced volume and function of the medial prefrontal cortex (mPFCx), hippocampus (HC) and amygdala (AMY) of RHA vs. RLA rats (Río-Álamos et al., 2017a, 2019). Finally, as it would be expected from a model with translational value, compared with RLAs, RHA rats also show more intense behavioral sensitization following the repeated administration of psychostimulants (Corda et al., 2005; Giorgi et al., 2005a, 2007) and enhanced vulnerability to drug abuse/addiction (Fattore et al., 2009), which are associated with a more robust mesolimbic dopaminergic tone (Giorgi et al., 1997, 2003, 2005b; Piras et al., 2003; Lecca et al., 2004; see “The Roman Rats as a Genetic Model of Vulnerability to Drug Addiction” section).

In recent years, it has become apparent that rather than a form of self-medication secondary to typical schizophrenia symptoms or medication side effects, substance abuse comorbidity is an independent primary disease symptom in schizophrenia (Chambers et al., 2001). Nevertheless, the limited availability of animal models of substance abuse comorbidity in psychotic disorders based on genetic selection is an important drawback in preclinical research on the impact of gene-environment interactions on such conditions. Here, we will survey experimental evidence supporting the view that the RHA rat line/strain may be used as a reliable animal model of genetically determined vulnerability to substance use comorbidity in schizophrenia in terms of face, construct, and predictive validity. Implications of these findings for investigations on the etiology and treatment of Impulsive Compulsive Spectrum Disorders are considered in the present review article.

## Tests and Models of the Impulsivity Trait and Impulsive Actions/Responses

Although preclinical and clinical studies have led to relevant advances in our knowledge of some psycho- and neuro-biological mechanisms underlying impulsivity and its involvement in several psychiatric disorders (Jupp and Dalley, 2014; Chase et al., 2017; Dalley and Robbins, 2017), the currently available treatments for impulsivity and impulsivity-related disorders have limited efficacy and application. Thus, rigorously validated animal models, which allow manipulation of experimental conditions that is not possible in humans, are critical to enable progress in our understanding of the psychological, neurobiological and genetic basis of deficits of impulse control and for the development of innovative therapies (Dalley et al., 2011; Hayward et al., 2016).

Of note, the assessment of the validity of animal models ought to address the three dimensions of face, construct, and predictive validity. Face validity refers to a phenomenological similarity between the model and the disorder being modeled. Construct validity implies that the model has a sound theoretical rationale and heuristic potential, that is, it can aid in discovering novel characteristics of the modeled condition. The concept of predictive validity implies that manipulations known to influence the pathological state (i.e., clinically effective treatments) should have similar effects in the model (Willner, 1984).

An important aspect of animal models involves the development of tests, tasks and/or measures that allow the evaluation of behavioral responses reflecting the human trait that one intends to model. In this regard, for example, the DRL-20, stop signal, delay discounting, schedule-induced polydipsia, 5C-SRT, and 5-choice continuous performance (5C-CP) tasks allow measuring impulsivity, impulsive responses and attention in laboratory rodents (Hayward et al., 2016; Dalley and Robbins, 2017). In addition, some of these models have the advantage of being completely translatable to humans (e.g., the stop signal paradigm, delay discounting, 5C-SRT and 5C-CP tests are used in both rodents and humans; Hayward et al., 2016; Dalley and Robbins, 2017).

Another significant aspect concerning animal analogs is the strategy employed to model impulsivity either from an “etiology-focused” or a “behavior to biology” perspective. Genetically engineered rats or mice and brain lesion-based models constitute experimental strategies for modeling impulsivity from an etiology-focused perspective. Notably, the etiological hypotheses are the main constraints of these models, which begin by assuming a given pathophysiological mechanism and then investigate the resulting phenotype (Jupp et al., 2013; Hayward et al., 2016). Conversely, the “behavior to biology” models use a different strategy, which starts by recreating or identifying an impulsive phenotype and then follows by investigating its neuropsychological underpinnings. Some of these preclinical models select groups of animals from a heterogeneous population on the basis of the different characteristics of a behavioral trait or behavioral response related with impulsivity. The Lister-Hooded rats selected for excessive premature (i.e., impulsive) responses in the 5C-SRT test (Robinson et al., 2009; Hayward et al., 2016; Dalley and Robbins, 2017) and the Wistar rats stratified for extreme values of schedule-induced polydipsia (Merchán et al., 2019, and references therein) are examples of these models of impulsive behavior. Other “behavior to biology” models use selective bidirectional breeding for a given phenotype and then systematically evaluate the underlying neurobiological mechanisms and associated traits. The latter are especially relevant since one would expect from a valid impulsivity model that such associated traits were related to the selected trait: for example, rats with an impulsive phenotype should be more vulnerable to addictive drug taking, compulsive drug seeking and relapse. Examples of these models are the low- and high-impulsive inbred rats (Belin et al., 2016), and the RHA and RLA rats (Escorihuela et al., 1999; Moreno et al., 2010; Tournier et al., 2013) that are the main focus of this review article.

## Coping Style, Behavioral Flexibility, Behavioral Inhibition and Impulsivity

The concept of coping style arises mainly from the field of research on stress and relates to how an organism deals, or tries to deal, with a threat. In general, it refers to consistent response profiles in the face of challenges that are similar across various situations and stable over time (Coppens et al., 2010). However, coping styles also extend their influence to non-threatening or non-aversive situations, such as appetitive conditions or tasks that involve some kind of conflict between actions that lead to worse consequences (e.g., a fast instrumental response that leads to an immediately available small reward) and actions that lead to a better outcome (e.g., a slow, delayed instrumental response that leads to a big reward). Two different styles of coping behavior, proactive and reactive, are considered in coping research. Typically, rodents with a proactive coping style show impulsive (i.e., premature and poorly conceived) responding profiles, display impaired behavioral flexibility (i.e., the ability to adapt responses to changing environmental demands) and attention, and are often novelty/incentive seekers (Coppens et al., 2010). Numerous studies indicate that the responses that characterize the proactive coping style are predominantly driven by internal mechanisms or routines (i.e., they are not much sensitive to changes in the environment), whereas reactive coping animals are much more dependent on the environment (Coppens et al., 2010). This difference in the sensitivity to changes in environmental conditions critically influences behavioral flexibility, which is generally higher in subjects characterized by a reactive coping style. For example, proactive coping RHA rats -that are also impulsive- are impaired at solving a spatial reversal learning task compared with their reactive coping RLA counterparts (Escorihuela et al., 1995; Río-Álamos et al., 2019). Thus, proactive coping styles in experimental animals and in humans are frequently positively associated with impulsivity, behavioral (cognitive) inflexibility and also with deficits in behavioral inhibition (for a review, see Coppens et al., 2010).

Behavioral inhibition is a trait related to the reactive coping style and anxiety. In this context, anxiety is operationally defined as the increased susceptibility to signals of punishment, non-reward and novelty, that leads to the expression of reactive coping responses, such as freezing or risk assessment in the face of a conflict between incompatible goals/responses (Gray and McNaughton, 2000). Conversely, proactive coping individuals tend to display disinhibited and impulsive responses/behavior under the same conditions (Gray and McNaughton, 2000; Avila and Torrubia, 2006; Coppens et al., 2010).

Notably, the RHA and RLA rat lines/strains display differential impulsivity and profound divergences in associated traits, such as coping style, behavioral inhibition, behavioral/cognitive flexibility, attention, cognitive ability (e.g., spatial learning, working memory) and novelty seeking, as described in the following section (Driscoll and Bättig, 1982; Driscoll et al., 1998, 2009).

## The Roman Rats: A Genetic Model of Differential Anxiety, Novelty Seeking, Impulsivity, Attention Deficits and Associated Traits

### Behavioral Inhibition and Anxiety

The Roman rat lines were established in Rome in the 1960s through bidirectional selection and outbreeding of Wistar rats showing very high (RHA) vs. extremely low (RLA) rates of acquisition of the two-way active avoidance response (Bignami, 1965, see “Introduction” section). Subsequently, the Swiss sublines RHA/Verh and RLA/Verh were derived from the original lines (Driscoll and Bättig, 1982) and other outbred sublines were established in the USA (Satinder, 1975), France (Willig et al., 1991; Castanon et al., 1995) and Cagliari, Italy (Giorgi et al., 2005b). In addition, two inbred Roman strains, RHA-I and RLA-I, generated through brother/sister mating of the respective Swiss sublines, were established in Barcelona and Germany (Schwegler et al., 1997; Driscoll et al., 1998; Escorihuela et al., 1999). It was soon noticed that the selection for two-way avoidance acquisition did not lead to rat lines primarily differing in some general process related to “learning ability.” In contrast, the Roman lines differ in the emotional responses displayed in stress or anxiety paradigms (reviewed by Driscoll and Bättig, 1982; Driscoll et al., 2009). Accordingly, there is ample experimental evidence showing that two-way active avoidance learning is blunted by fear/anxiety (Gray, 1980; Fernández-Teruel et al., 1991b; Gray and McNaughton, 2000; Vicens-Costa et al., 2011; LeDoux, 2015; LeDoux et al., 2017; Fernández-Teruel and Tobeña, 2018). In fact, RHA rats learn faster and more effectively than their RLA counterparts to avoid the electric shock in the two-way active avoidance test, whereas RLA rats perform better than RHA rats in some operant and spatial memory tasks (Zeier et al., 1978; Nil and Bättig, 1981; Driscoll and Bättig, 1982; Willig et al., 1991).

In parallel, early studies on emotional aspects showed that RLA rats were more sensitive to classical Pavlovian aversive conditioning than their RHA counterparts (Imada, 1972) and displayed more robust hormonal responses (i.e., increments in the plasma concentrations of corticosterone, prolactin, and ACTH) to several types of acute psychological and physical stressors (Gentsch et al., 1981, 1982). These neuroendocrine findings have been replicated in many subsequent studies (Castanon et al., 1992; Castanon and Mormède, 1994; Steimer et al., 1998; Steimer and Driscoll, 2003; Carrasco et al., 2008; Díaz-Morán et al., 2012; Río-Álamos et al., 2017a,b).

Anxiety response profiles are markedly different between RHA and RLA rats, as evidenced in experimental procedures involving unconditioned or conditioned conflict. Among the former, some of the earliest demonstrations suggesting between-line differences in anxiety were the increased defecation and reduced exploratory activity observed in RLA rats in the open field test (Gentsch et al., 1981, 1982) and their avoidance of the illuminated center of the hexagonal tunnel maze (Driscoll and Bättig, 1982; Martin et al., 1982). These results have been corroborated in numerous studies (Fernández-Teruel et al., 1991a, 1992a,b, 1994, 1997, 2002b; Escorihuela et al., 1997, 1999; Steimer and Driscoll, 2003; Estanislau et al., 2013; Tapias-Espinosa et al., 2018) and extended to other novelty-based, unconditioned conflict conditions, such as the hyponeophagia test, the light/dark box, the elevated plus-maze and the elevated zero-maze, in which RLA rats consistently display higher levels of behavioral inhibition and anxiety responses, which include avoidance of open/illuminated spaces, freezing, reduced exploratory activity and increased self-grooming (Ferré et al., 1995; Steimer et al., 1998; Escorihuela et al., 1999; Fernández-Teruel et al., 2002b; Steimer and Driscoll, 2003; Estanislau et al., 2013; Río-Alamos et al., 2015; Río-Álamos et al., 2017a,b).

Moreover, in procedures involving Pavlovian aversive conditioning or conditioned approach-avoidance conflict, RLA rats display, compared to their RHA counterparts, increased conditioned freezing (Imada, 1972; López-Aumatell et al., 2009a; Martínez-Membrives et al., 2015), enhanced shock-induced suppression of drinking (Ferré et al., 1995; Corda et al., 2018) and more pronounced fear-potentiated acoustic startle (Schwegler et al., 1997; Yilmazer-Hanke et al., 2002; López-Aumatell et al., 2009a,b). The enhanced behavioral inhibition of RLA rats is also observed in instrumental procedures of frustrative reward omission (i.e., extinction) or reward down-shift (Torres et al., 2005; Rosas et al., 2007; Gómez et al., 2009b; Coppens et al., 2012, 2013; Manzo et al., 2014b). Finally, another example of the reactive (i.e., behavioral inhibition) vs. proactive coping styles in the Roman lines/strains is that, in the forced swimming test, an experimental model that is extensively used for the screening of antidepressant drugs (Porsolt et al., 1977), RLA rats display fewer active movements aimed at escaping from the water cylinder (i.e., struggling) and more immobility than RHA rats (Piras et al., 2010, 2014; Díaz-Morán et al., 2012). Moreover, either the subacute or chronic treatment with the antidepressants desipramine, chlorimipramine or fluoxetine significantly decrease immobility in RLA, but not RHA rats (Piras et al., 2010, 2014), supporting the predictive validity of the model.

Notably, it has been reported that GABA-related anxiolytic drugs decrease behavioral inhibition (or anxiety) of RLA, but not RHA rats, in some of the above mentioned conflict-based tests (Martin et al., 1982; Driscoll and Stübi, 1985; Fernández-Teruel et al., 1991c; Steimer and Driscoll, 2003; Torres et al., 2007), whereas the anxiogenic drug pentylenetetrazol, a blocker of the chloride channel of the GABA-A receptor, enhances behavioral inhibition (i.e., anxiety) in the Vogel’s conflict test predominantly in the RLA line (Corda et al., 2018). Of relevance in this context is the finding that the function of the GABA-A receptor (assessed by means of the GABA-stimulated ^36^Cl^−^ uptake and GABA-stimulated [^3^H]-Zolpidem binding) is reduced in the whole cerebral cortex of RLA vs. RHA rats (Giorgi et al., 1994; Bentareha et al., 1998), which may contribute to the divergent behavioral inhibition and emotional responses that distinguish RHA from RLA rats.

Gray and McNaughton (2000) and McNaughton and Corr (2004) have proposed that behavioral inhibition, a main expression of cognitive anxiety, is a consequence of the activation of the “Behavioral Inhibition System,” whose neuroanatomical substrate is the septo-hippocampal system (SHS). The activity of the SHS, in close interplay with the AMY and related circuitry, seems to be responsible for the appearance of some species-specific inhibitory responses, such as freezing or risk-assessment, in the face of situations in which there is a conflict between incompatible goals/responses or uncertainty (Gray and McNaughton, 2000). That is, the activation of the SHS, as well as the AMY in more specific situations, leads to responses that characterize states of anxiety and/or fear (Gray and McNaughton, 2000; LeDoux, 2015; see also Fernández-Teruel and Tobeña, 2018). Activation of the central GABAergic system by anxiolytic benzodiazepines and other GABA-mimetic drugs decreases the activity of the SHS, and thus reduces behavioral inhibition in conflict conditions (Gray and McNaughton, 2000). It is noteworthy that, compared with their RHA counterparts, RLA rats display a more pronounced neural activation of the HC and AMY, as reflected by increased *c-fos* expression, when exposed to different novelty-based conflict situations (Meyza et al., 2009). Accordingly, the volume of the HC and AMY is larger (Río-Álamos et al., 2017a), and the neuronal density is higher in both brain areas of RLA vs. RHA rats (Garcia-Falgueras et al., 2012; Gómez et al., 2009c). In addition, the activity of the phospholipase C signaling cascade is higher in the HC of RLA vs. RHA rats (Sallés et al., 2001).

Collectively, the experimental findings reviewed above support the view that RHA rats are less anxious and more behaviorally disinhibited than RLA rats and other rat strains/stocks used as external controls, such as Wistar rats or the “*National Institutes of Health Heterogeneous Rat Stock”* (NIH-HS) rats (Gómez et al., 2009a; López-Aumatell et al., 2009b; Díaz-Morán et al., 2012; Estanislau et al., 2013; Martínez-Membrives et al., 2015).

### Sensation Seeking and Impulsivity

As mentioned in “Coping Style, Behavioral Flexibility, Behavioral Inhibition and Impulsivity” section, the proactive coping style of RHA rats is associated with behavioral disinhibition and novelty/sensation seeking. In this regard, the relative behavioral disinhibition of RHA vs. RLA rats is also apparent in tests measuring the preference for novelty which involves some degree of “approach-avoidance” conflict and are considered to capture more specifically the sensation/novelty seeking (SNS) trait. Such tests include the novel object exploration test, the “Y” maze, familiar vs. unfamiliar spaces, which include novel objects and novel/unknown spatial distributions (Pisula, 2003), and the hole-board test with unfamiliar objects under the holes (File and Wardill, 1975). In these tests, compared with the RLA strain and, in some measures/tests, also with the outbred NIH-HS strain, RHA rats consistently display more intense exploration of the novel/unfamiliar objects or spaces (Fernández-Teruel et al., 1992b, 1997; Escorihuela et al., 1999; Pisula, 2003; Estanislau et al., 2013; Manzo et al., 2014a; Cuenya et al., 2016).

The SNS trait in humans, cats and rats is characterized by high levels of exploratory and risk-taking behaviors and is commonly associated with impulsiveness and a tendency to experiment with drugs of abuse (Bardo et al., 1996; Siegel, 1997). SNS cats, for example, show increased exploratory behavior and lowered efficiency in bar pressing responses during an inhibitory DRL task (i.e., an appetitive instrumental conditioning paradigm in which reward is obtained only following low response rates; Saxton et al., 1987a,b). Importantly, a similar behavior has been reported in RHA rats (Zeier et al., 1978).

Regarding the neurophysiological basis of SNS, an interesting early finding is that, like SNS humans and cats, RHA rats are visual evoked potential augmenters at the brain cortical level, whereas RLA rats (as well as the Wistar rats used for comparison) are visual evoked potential reducers (Siegel and Driscoll, 1996; Siegel et al., 1996; Siegel, 1997).

Studies in humans have shown that behavioral disinhibition is a component of sensation seeking and that this trait is moderately correlated with impulsivity. Thus, although sensation seeking and impulsivity are different traits, they show a significant positive association. This finding has prompted the development of composite psychometrical measures of “impulsive sensation seeking,” using psychometrical instruments that combine both traits into a single construct (Zuckerman, 1996; Roberti, 2004; Magid et al., 2007).

The RHA rats encompass many characteristics that are consistent with a model of “impulsive sensation seeking.” Part of the evidence reviewed above, such as the deficit of RHA rats in the DRL20 paradigm, in which they are less able to inhibit irrelevant responses than their RLA counterparts (Zeier et al., 1978), or the behavior of RHA vs. RLA rats in some learning tasks in several types of mazes (Nil and Bättig, 1981; Driscoll and Bättig, 1982; Willig et al., 1991), had already suggested that deficits of impulse control in RHA rats could play a role in their deficient performance in those tasks. Accordingly, it has been reported that inhibitory control is impaired in RHAs compared with RLA rats, as shown by an increased number of premature responses made by the former in the 5-CSRT task (Moreno et al., 2010). In a delay discounting task, RHA rats also display enhanced impulsive choice and prefer the immediate and smaller reward as opposed to a delayed larger reinforcement (Moreno et al., 2010). In addition, in the procedure of schedule-induced polydipsia, the RHA strain shows stronger acquisition of the adjunctive responses, and a higher asymptotic response level of both water intake and licking responses than RLA rats (Moreno et al., 2010; see also Klein et al., 2014; Merchán et al., 2019).

Consistent with the contention that they represent an impulsive phenotype, RHA rats display a higher frequency of lever pressing than their RLA counterparts during the acquisition phase in an unpredictable operant conditioning paradigm and during the extinction phase upon appetitive instrumental conditioning (Gómez et al., 2009a; Moreno et al., 2010; Coppens et al., 2012, 2013; Klein et al., 2014). Moreover, RHA rats are more perseverant than RLAs at pressing the active lever during the extinction phase of an intravenous cocaine self-administration program (Fattore et al., 2009; see “The Roman Rats as a Genetic Model of Vulnerability to Drug Addiction” section). Thus, the RHA phenotype includes characteristics of both the SNS and impulsivity traits.

The absence of strain-dependent differences in home cage activity (Meyza et al., 2009; Estanislau et al., 2013) as well as the finding that both Roman lines display similar lever press responding during the acquisition of an appetitive operant task under a fixed ratio-1 (Coppens et al., 2013), argues against the possibility that the distinct impulsive responding of the two lines/strains is due to differences in motor activity.

Notably, although RHA rats show a more impulsive behavioral profile than their RLA counterparts, it is clear that they are capable of displaying response inhibition under threatening conditions; thus, RHA rats show high levels of conditioned freezing behavior, either when exposed to a cue (López-Aumatell et al., 2009a) or a conditioned context during Pavlovian or operant aversive conditioning (López-Aumatell et al., 2009a,b; Díaz-Morán et al., 2012; Martínez-Membrives et al., 2015).

A large body of experimental evidence has established that the mesolimbic dopamine (DA) system is a key component of the neural circuitry mediating brain reward (Wise, 2002). This system consists of DA neurons in the ventral tegmental area (VTA) of the mesencephalon that project to forebrain regions such as the nucleus accumbens (Acb), mPFCx, AMY, and HC. Activation of the mesolimbic DA system plays a pivotal role in sensation seeking behavior (Bardo et al., 1996) and in the reinforcing effects of drugs of abuse (Wise, 2002; Pierce and Kumaresan, 2006; see “The Roman Rats as a Genetic Model of Vulnerability to Drug Addiction” section).

It has been shown that the density of D2 DA auto-receptors (D2-autoR) is lower in the substantia nigra/VTA of RHA vs. RLA rats (Tournier et al., 2013), consistent with reports relating SNS with low midbrain DA D2-autoR density in humans (Zald et al., 2008). Moreover, the density of postsynaptic DA D2/D3 receptors is lower in the striatum and nucleus accumbens (Acb) of RHA vs. RLA rats and it has been shown that striatal DA D2 receptor availability is inversely correlated with novelty seeking behavior of Roman rats in the hole-board test (Tournier et al., 2013). Importantly, sensation seeking in humans has also been negatively associated with striatal D2 receptor availability (Gjedde et al., 2010). Taken together, the above mentioned findings and the effects of dopamine-mimetic psychostimulants on striatal D2 receptor availability and on the extracellular concentrations of DA in the terminal fields of the mesolimbic dopaminergic projections support the view that the functional tone of the mesolimbic dopaminergic system is more intense in impulsive sensation seekers such as RHAs than RLA rats (Giorgi et al., 2007; Tournier et al., 2013).

In studies using rats selected for impulsivity, i.e., behaviorally separated in extreme “high impulsive” (HI) vs. “low impulsive” (LI) groups according to their levels of premature responding in the 5-CSRT task, it was found that HI animals had decreased D2/D3 receptor availability in the Acb, and D2/D3 receptors negatively predicted impulsive behavior (Dalley et al., 2007; see also Barlow et al., 2018). This is consistent with the aforementioned findings in the Roman rats and with a similar D2/D3 receptor profile found in ADHD patients (Buckholtz et al., 2010; Ghahremani et al., 2012).

Finally, a recent fMRI study in a large sample of healthy and distressed humans has shown that the activity of the left ventrolateral prefrontal cortex and bilateral ventral striatum, including the Acb, during an “uncertain reward expectancy” paradigm is positively related with impulsive sensation seeking (Chase et al., 2017). This is also in line with the proposal of an enhanced functional tone of the mesolimbic dopaminergic pathway in impulsive sensation seekers like RHA rats (Giorgi et al., 2007).

To sum up, RHA rats show considerable similarities with impulsive and sensation seeking humans regarding SNS, impulsivity, visual evoked potentials, and the underlying mesolimbic DA transmission-related parameters. These findings highlight the face and construct validity of RHA rats as a genetic model of impulsivity and SNS.

## RHA Rats as a Model of Deficits in Attentional Processes, Cognitive Impairments and Other Features Relevant for Schizophrenia Research

As mentioned previously, a proactive coping style is often associated with impairments in attention and cognitive flexibility, as well as heightened impulsivity (for a review see Coppens et al., 2010). These traits have in turn been associated with schizophrenia in patients (Brown et al., 2018; Ho et al., 2018). Thus, using both psychometric impulsivity scales and the delay discounting task (a measure of action planning and impulse control in which higher rates of reward discounting, due to the preference of smaller and immediate rewards over delayed and larger ones, reflect enhanced impulsivity), it has been shown that impulsivity measures are positively associated in a consistent manner with schizophrenia, schizoaffective disorder, and schizophrenia risk in unaffected biological relatives of patients (Ahn et al., 2011; Brown et al., 2018; Ho et al., 2018). In addition, the presence of heightened impulsivity in schizophrenia is paralleled by impairments in working memory (Ahn et al., 2011; Brown et al., 2018).

Therefore, we considered of interest to investigate whether RHA rats would also display cognitive and attentional impairments and other schizophrenia-relevant phenotypes. In this context, more than two decades ago it was demonstrated that there were marked differences in spatial cognitive abilities between the Roman rat lines. Thus, spatial reference learning (i.e., place learning) in the Morris water maze was impaired in RHA rats (Driscoll et al., 1995; Escorihuela et al., 1995). Moreover, RHA rats also were impaired in a reversal place task, suggesting a blunted behavioral/cognitive flexibility relative to their RLA counterparts (Escorihuela et al., 1995; Fernández-Teruel et al., 1997). These early findings have been replicated and extended by showing that RHAs also exhibit spatial working memory deficits, as compared with both RLA rats and the genetically heterogeneous NIH-HS rats (Willig et al., 1991; Aguilar et al., 2002; Oliveras et al., 2015, 2016).

It is noteworthy in this context that the impairments in working memory of schizophrenic patients may be due, at least in part, to alterations in cortical GABAergic inhibitory transmission. Accordingly, it has been shown that, in individuals with schizophrenia, a deficiency in brain derived neurotrophic factor- (BDNF-) mediated signaling combined with decreased levels of the GABA synthetizing enzyme, GAD67, results in reduced inhibitory neurotransmission of GABA neurons (i.e., basket cells) in the dorsolateral prefrontal cortex (Lewis et al., 2005; Kimoto et al., 2014). This deficiency in GABA-mediated transmission leads to a decreased inhibition of pyramidal neurons that have been hypothesized to contribute to altered gamma oscillations and impaired working memory in schizophrenia (Lewis et al., 2005). In view of the above findings it would be worthwhile to evaluate GABAergic transmission in the mPFCx (corresponding to the dorsolateral frontal cortex of the human brain) of RHA and RLA rats, since our previous results concerning GABA-stimulated ^35^Cl^−^ uptake were performed in the whole cerebral cortex (Giorgi et al., 1994; Bentareha et al., 1998; see “The Roman Rats: A Genetic Model of Differential Anxiety, Novelty Seeking, Impulsivity, Attention Deficits and Associated Traits” section).

LI and PPI of the startle response are attention-related processes that are impaired in schizophrenia and other diseases, such as bipolar disorder (Gray et al., 1991; Swerdlow et al., 1994, 1996; Cromwell et al., 2008; Lubow and Weiner, 2010; Kohl et al., 2013). The procedures used to evaluate LI and PPI are similar in rodents and humans, and the performances in both paradigms are currently considered as endophenotypes of schizophrenia (Gray et al., 1991; Powell and Miyakawa, 2006; Lubow and Weiner, 2010; Jones et al., 2011; Swerdlow and Light, 2016). Notably, RHA rats display deficient LI of the two-way active avoidance response compared with Sprague-Dawley rats (Fernández-Teruel et al., 2006). Likewise, compared with RLAs, RHA rats show impaired LI of the fear-potentiated startle (Esnal et al., 2016).

In line with these strain-related differences in LI, we have shown in a series of studies that RHA rats display clear and consistent deficits of PPI of the acoustic startle response compared with RLAs (Del Rio et al., 2014; Oliveras et al., 2015, 2016, 2017; Río-Alamos et al., 2015; Río-Álamos et al., 2017a,b, 2019; Tapias-Espinosa et al., 2018, 2019) and that PPI deficits predict spatial working memory impairments (Oliveras et al., 2015). Altogether, the above mentioned cognitive and attentional profiles of RHA vs. RLA rats suggest that the former may represent a model of schizophrenia-relevant features. This is further supported by the increased mesolimbic/mesostriatal dopaminergic functional tone of RHA rats (Giorgi et al., 2007; Tournier et al., 2013), their enhanced sensitivity to the locomotor and DA releasing effects of acute and chronic psychostimulants (Corda et al., 2005; Giorgi et al., 2005a,b, 2007; Guitart-Masip et al., 2008), and by the finding that, relative to RLA rats, RHAs exhibit some impairments in social behavior (Coppens et al., 2012; Del Rio et al., 2014).

It is noteworthy that PPI of the acoustic startle is further impaired by the non-selective DA D1/D2 receptor agonist apomorphine and improved by the DA D2/D3 receptor antagonist haloperidol in RHAs but not RLA rats (Oliveras et al., 2017).

Two additional lines of evidence support the view that RHA rats may be considered as a putative model of schizophrenia-relevant symptoms/features. Firstly, social isolation rearing induces deleterious effects on PPI, anxiety, and spatial reference memory only in RHA rats (Oliveras et al., 2016). Second, in Sprague-Dawley rats, bilateral neonatal lesions of the ventral hippocampus (NVHLs), a neurodevelopmental model of schizophrenia, result in enhanced locomotor activation and stereotypies in adult rats upon the acute administration of amphetamine (Lipska and Weinberger, 2000). Moreover, in adult NVHL rats, a challenge with amphetamine elicits an augmented increase in DA output in the core (AcbCo) and, at the same time, an attenuated dopaminergic response in the shell (AcbSh) of the Acb, as compared with shams (Corda et al., 2006). Importantly, the effects of the amphetamine challenge in this neurodevelopmental model of schizophrenia are closely reminiscent of those observed in psychostimulant-sensitized RHA rats (Giorgi et al., 2005b, 2007; see also “The Roman Rats as a Genetic Model of Vulnerability to Drug Addiction” section).

Notably, an interesting recent development is the finding that, compared with their RLA counterparts, RHA rats display a severe disruption of the dimeric metabotropic glutamate-2 (mGlu2)/5-HT2A receptor complex consisting in a dramatic reduction of the cortical, hippocampal and striatal density of mGlu2 receptors, associated with an increase of  5-HT2A receptor density in the frontal cortex (Klein et al., 2014; Fomsgaard et al., 2018). This molecular profile is reminiscent of what has been found in the cortex of drug-free schizophrenic patients (González-Maeso et al., 2008). Accordingly, it has been shown that the dimeric mGlu2/5-HT2A receptor complex is critically involved in the pharmacologic effects of atypical antipsychotics; thus, clozapine does not decrease locomotion in rodents with a disruption of the dimeric mGlu2/5-HT2A receptor complex (González-Maeso et al., 2008; Kurita et al., 2012). Therefore, the alterations observed in this complex in RHA rats may at least partly account for some of the phenotypical schizophrenia-related traits observed in this strain and the almost complete lack of enhancing effect of clozapine on either the baseline or MK-801-impaired PPI performance (Oliveras et al., 2017).

On the other hand, serotonin transmission has also been related also to impulsivity (Dalley and Robbins, 2017). Thus, in RHA rats, but not RLAs, there is a positive correlation (*r* = 0.94, *p* = 0.02) between 5-HT2A receptor density and premature responses in the 5-CSRT task (Klein et al., 2014). This is consistent with findings from studies in rodents and humans in which the pharmacological blockade of the 5-HT2A receptor produces reductions of several types of impulsive responses (Klein et al., 2014; Dalley and Robbins, 2017). These findings suggest that 5-HT2A receptors are involved in impulsivity and, more specifically, that they may facilitate at least some forms of impulsive behavior (reviewed by Klein et al., 2014; see also Dalley and Robbins, 2017).

Altogether, the above reviewed behavioral, pharmacological, molecular and neuroanatomical profiles point to RHA rats as a model exhibiting several phenotypical characteristics relevant for impulsivity, sensation seeking, and schizophrenia research. These phenotypes include attentional and cognitive impairments, deficits in social behavior, premature responding and waiting impulsivity, enhanced mesolimbic dopaminergic activity and schizophrenia-like alterations of cortical mGlu2 and 5-HT2A receptors.

Finally, it has recently been shown that, compared with their RLA counterparts, RHA rats exhibit significant reductions of the volumes of the mPFCx and HC [measured by means of magnetic resonance imaging (MRI)] as well as dramatically enlarged lateral ventricles (Río-Álamos et al., 2017a, 2019). In addition, PPI-elicited neural activity of the prefrontal cortex (as measured by *c-fos* activation) is lower in RHA than RLA rats (Tapias-Espinosa et al., 2019). These neuroanatomical and functional alterations are reminiscent of those observed in schizophrenic patients and provide further support to the contention that RHA rats may constitute a genetic model of schizophrenia-relevant features with face, construct, and predictive validity (reviewed by Río-Álamos et al., 2019).

## The Roman Rats as a Genetic Model of Vulnerability to Drug Addiction

A combination of environmental and genetic factors underlies the vulnerability to develop a drug addiction (Nestler, 2001, 2000; Kreek et al., 2005a). Accordingly, interacting with the direct effects of abused drugs, environmental and genetic factors may induce the transition from casual, intermittent drug use to regular intake, the progression from abuse to compulsive drug seeking and taking (i.e., addiction), and the propensity for repeated relapse even after a long-term drug-free state (Kreek et al., 2005a,b; Everitt and Robbins, 2016).

In this section, we examine experimental evidence supporting the view that the Roman lines represent a valid model to investigate how and to what extent the genetic background influences the neural and behavioral traits involved in the major features of the individual vulnerability to addiction, including high sensitivity to the acute effects of drugs of abuse, the development of sensitization upon repeated administration, and the propensity to self-administer addictive drugs.

Early work in the 1980s–90s provided evidence suggesting that the behavioral patterns distinguishing the Roman rat lines may be mediated, at least in part, by differences in the functional properties of the mesolimbic and mesocortical dopaminergic projections. Thus, it was shown that: (i) various stressors activated the mesocortical dopaminergic pathway in RHAs but not RLA rats (D’Angio et al., 1988; Giorgi et al., 2003); (ii) RHA rats showed a faster turnover rate of DA in the caudate nucleus (Driscoll et al., 1990) and displayed more intense stereotypies and yawning than RLA rats upon acute challenge with apomorphine (Driscoll et al., 1985; Gimenez-Llort et al., 2005; Sanna et al., 2013); and (iii) the density of DA D1 receptors in the Acb was higher in RHA vs. RLA rats (Giorgi et al., 1994; Guitart-Masip et al., 2006).

The mesolimbic dopaminergic pathway has long been considered to play a major role in mediating the reinforcing effects of psychostimulants, opiates, nicotine, ethanol, and cannabinoids (Pierce and Kumaresan, 2006). Thus, the above-mentioned differences between the Roman lines, together with differences in sensation seeking and impulsive behavior, prompted us to investigate how drugs of abuse affect behavior and central DA function in both rat lines and whether there are differences in addiction liability between them. In this context, and in line with the hypothesis that RHA rats would be more responsive to drugs of abuse than their RLA counterparts, we found that acutely administered morphine and the psychostimulants cocaine or amphetamine elicit larger increments in DA output, as measured by brain microdialysis, in the AcbSh than the AcbCo of RHA rats, whereas such difference is not present in RLA rats (Lecca et al., 2004). Moreover, the three drugs induce a higher increase of locomotor activity in the former line (Giorgi et al., 1997, 2005a,b; Piras et al., 2003; Lecca et al., 2004; reviewed by Giorgi et al., 2007).

The line-related differences in the effects of acute morphine and psychostimulants on the mesolimbic dopaminergic transmission suggested that the Roman lines also may differ in the responsiveness of their neural circuits of reward to other appetitive stimuli. Accordingly, compared with RLA rats, RHAs have been shown to display higher preference for ethanol and larger increases in DA output in the AcbSh after acute ethanol intake (Fernández-Teruel et al., 2002a; Manzo et al., 2012, 2014a; Corda et al., 2014). Furthermore, RHA rats also display higher preference for saccharin solutions (Razafimanalina et al., 1996; Fernández-Teruel et al., 2002a) and palatable food (Giorgi et al., in preparation) and show more intense sexual motivation than their RLA counterparts (Sanna et al., 2017, 2019). Of note, palatable food intake and sexual activity are associated with a larger increment in accumbal DA output in RHA than RLA rats (Giorgi et al., in preparation; Sanna et al., 2017, 2019).

The above findings prompted us to study whether the Roman lines also differ in the propensity to develop behavioral sensitization upon repeated administration of drugs of abuse. Behavioral sensitization is characterized by a progressive increase in the intensity of locomotor activation and stereotypes upon repeated exposure to a constant dose of a drug, such as psychostimulants and opiates, or by a greater response on re-challenge with a lower dose of drug than used in the initial chronic-intermittent exposure (Segal and Kuczenski, 1987; Pierce and Kalivas, 1997; Vanderschuren and Kalivas, 2000). This increased response is believed to reflect long lasting adaptations in neural circuits involved in motivation and reward (Nestler, 2001; Robinson and Berridge, 2001; Everitt and Wolf, 2002; Li et al., 2004). Accordingly, repeated exposure to psychostimulants enhances the incentive motivational properties of addictive drugs, facilitates drug self-administration (Horger et al., 1990; Piazza et al., 1990; Mendrek et al., 1998; Lorrain et al., 2000), and potentiates the responses to conditioned rewards (Shippenberg and Heidbreder, 1995; Taylor and Horger, 1999; for a review, see Giorgi et al., 2007). The locomotor effects observed following the repeated administration of psychostimulants or morphine consistently indicate that the Roman rat lines differ markedly in the susceptibility to develop behavioral sensitization (Piras et al., 2003; Corda et al., 2005; Giorgi et al., 2005a,b, 2007): RHA rats displayed a significant increment in locomotor activity after a challenge dose of each drug given several days/weeks upon completion of the repeated drug treatment whereas no enhanced locomotor activity in response to the challenge drug dose was observed in RLA rats (Piras et al., 2003; Corda et al., 2005; Giorgi et al., 2005a, 2007; Guitart-Masip et al., 2008; Tournier et al., 2013).

Moreover, brain microdialysis assays revealed that the challenge with cocaine, amphetamine or morphine elicited larger increases in DA output in the AcbCo of RHA than RLA rats that had respectively received repeated doses of cocaine, amphetamine or morphine (Piras et al., 2003; Giorgi et al., 2005b, 2007). In parallel, and only in RHA rats, the drug challenges induced an attenuated dopaminergic response in the AcbSh. It is remarkable that the above mentioned repeated drug treatment schedules, which induced behavioral sensitization only in RHA rats, produced concomitant neural adaptations in the AcbCo and AcbSh exclusively in this rat line (Giorgi et al., 2007).

Behavioral and neurochemical sensitization to abused drugs is thought to play a key role in several addiction-related features, such as compulsive drug-seeking and the long-lasting vulnerability to relapse (see Fattore et al., 2009 and references therein). Therefore, we considered of interest to investigate the susceptibility of RHA and RLA rats to develop drug self-administration. To this aim, we assessed the acquisition, maintenance, extinction, reinstatement and reacquisition of intravenous self-administration of cocaine in RHA and RLA rats in an operant FR-1 task (Fattore et al., 2009). It was found that RHA rats displayed substantially more frequent lever-press operant responding than their RLA counterparts during all the phases of cocaine self-administration.

The persistently higher frequency of active lever responding of RHA vs. RLA rats during the maintenance phase of cocaine self-administration could not be attributed to a generalized rate effect in RHA rats since the inactive lever responding was not increased relative to RLA rats. Furthermore, extinction tests were conducted in the absence of cocaine reinforcement, and yet cocaine-seeking responses at the active lever in RHA rats were more frequent relative to RLAs and required almost twice as many test sessions to reach extinction criteria (Fattore et al., 2009).

After 3 weeks of withdrawal, when RHA and RLA rats had extinguished to similar low response levels, non-contingently administered cocaine injections induced significantly more frequent responses at the active lever in RHAs relative to RLA rats, whereas saline injections were without effect in both lines. Collectively, these results suggest that RHA rats are more susceptible than RLAs to relapse to cocaine seeking, whether induced by exposure to the cocaine-associated context (i.e., extinction), or to a priming injection of cocaine (i.e., reinstatement). In addition, these findings support the contention that, in RHA rats, behavioral sensitization may be involved in relapse-related processes (Fattore et al., 2009). Importantly, the AcbCo has been implicated in the reinstatement of cocaine-seeking induced by the administration of a priming dose of the drug or by exposure to drug-related cues (Ito et al., 2004; Hollander and Carelli, 2007). This experimental evidence is consistent with the view that the AcbCo and its afferent glutamatergic projections originating in the mPFCx play a key role in cocaine-induced relapse (McFarland et al., 2003). Accordingly, as mentioned above, the functional tone of the dopaminergic transmission is markedly increased in the AcbCo of RHA rats behaviorally sensitized to cocaine (Giorgi et al., 2007).

Besides the central role of the mesolimbic dopaminergic projection to the Acb, other brain areas and neural circuits may be involved in the susceptibility of RHA rats to develop cocaine seeking and taking upon chronic exposure to the same drug. For instance, a dysfunction of the frontocortical projections to limbic nuclei, which is thought to be a consequence of long-term exposure to drugs of abuse, may lead to a deficient inhibition of inappropriate/irrelevant responses during operant extinction or in the presence of drug-paired stimuli (Jentsch and Taylor, 1999). Accordingly, in RHA rats behaviorally sensitized following chronic treatment with cocaine, but not in their RLA counterparts, the dopaminergic transmission in the mPFCx is markedly reduced (Giorgi et al., in preparation). Furthermore, compared with their RLA counterparts, RHA rats show a smaller frontocortical volume (Roberti, 2004; Río-Álamos et al., 2019) and a reduced activation of the mPFCx upon exposure to PPI testing and a variety of novelty-related situations (Meyza et al., 2009; Río-Álamos et al., 2019; Tapias-Espinosa et al., 2019).

Substance use comorbidity is a frequent event in many psychiatric disorders and is particularly prevalent in schizophrenic populations (Selzer and Lieberman, 1993). Thus, dual diagnosis in schizophrenia is associated with a remarkably high prevalence of cocaine, amphetamine, alcohol, cannabis, and nicotine use (DeQuardo et al., 1994; Buckley, 1998; Dalack et al., 1998; Dixon, 1999).

A widely held neurobiological explanation for substance use comorbidity in schizophrenia is the self-medication hypothesis, which postulates that patients use addictive drugs to relieve aversive disease symptoms or the adverse side effects of medication. Hence, this hypothesis posits that susceptibility to drug abuse is a reaction to the psychotic disorder or medication side effects, and thus represents a secondary symptom (Buckley, 1998; Dalack et al., 1998; Krystal et al., 1999). In contrast, experimental evidence accumulated in the last years supports the view that the pathologic substrate of schizophrenia may contribute to the susceptibility to addiction by facilitating the functional activity of the neural circuitry that mediates positive reinforcement (Chambers et al., 2001). Two key lines of evidence support this primary addiction hypothesis: (i) the putative neuropathology underlying schizophrenia involves alterations in neural circuits that regulate positive reinforcement, incentive motivation, novelty seeking, behavioral inhibition, and addictive behavior as well as schizophrenia-relevant attentional/cognitive impairments (Chambers et al., 2001 and references therein); and (ii) experimental manipulations that model neuropathologic and behavioral aspects of schizophrenia in animals (e.g., the NVHL model, Lipska and Weinberger, 2000) also facilitate positive reinforcement and the incentive motivational effects of rewarding stimuli (Chambers and Self, 2002). An implicit feature of this hypothesis is that both the schizophrenia syndrome and vulnerability to addiction are primary disease symptoms, each directly caused by common neuropathologic substrates.

Most important, the results reviewed herein show that RHA rats include behavioral and neurochemical traits related with both, schizophrenia and addiction. Therefore, RHA rats may represent a promising model of substance use comorbidity with face, construct, and predictive validity.

## An Integrated Perspective

Collectively, the findings reviewed herein provide a detailed account of the major traits that distinguish RHA from RLA rats, from coping style, impulsivity, and behavioral inhibition through attention and cognitive ability to novelty seeking, drug seeking, mesocorticolimbic dopaminergic transmission, and schizophrenia-relevant behaviors.

**Figure 1:**
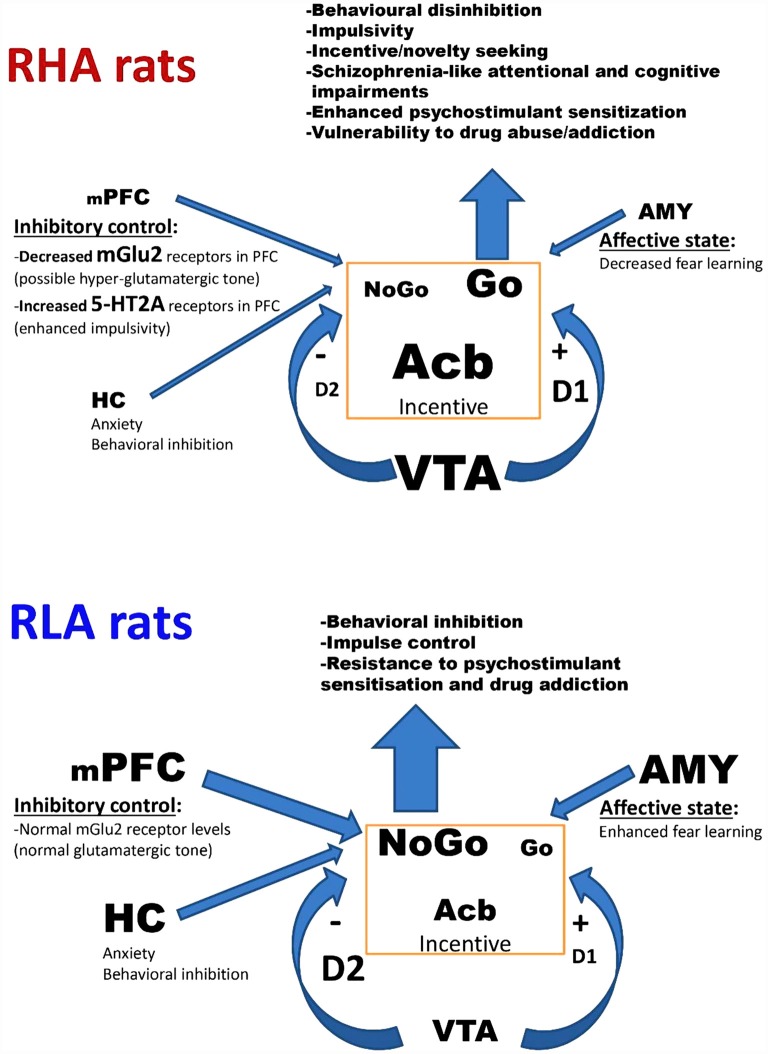
Simplified model of the functions of the mesocorticolimbic dopaminergic circuit and its modulation by limbic and cortical areas in relation to their differential involvement in behavioral inhibition/disinhibition, impulsivity, schizophrenia-like features, and drug seeking/addiction in Roman high- (RHA) and low-avoidance (RLA) rats. In this schematic diagram, the arrow thickness and character size represent the intensity of the functional tone or the receptor density of the corresponding brain area or neural circuit, respectively. The lower neuronal activity and volume of the medial PFC (mPFC), the HC, and the AMY, as well as the increased density of 5-HT2AR and the markedly decreased density of mGlu2R in RHA vs. RLA rats is consistent with the possibility of a hyper-functional glutamatergic cortical system that would in turn lead to an increased functional tone of the mesolimbic dopaminergic system and a decreased functional tone of the meso-cortical dopaminergic system in RHA rats. Collectivelly, the lowered function of the mPFC, the HC, and the AMY, along with the increased functional tone of the mesolimbic dopaminergic system of RHA rats favor behavioral disinhibition, impulsive actions/responses, attentional/cognitive deficits and vulnerability to drug addiction. Modified from Probst and van Eimeren (2013). Abbreviations: mPFC, medial prefrontal cortex; HC, hippocampus; AMY, amygdala; Acb, nucleus accumbens; VTA, ventral tegmental area; mGlu2R, metabotropic glutamate type 2 receptors; 5-HT2AR, serotonin type 2A receptors; D2R, dopamine type 2 receptors; D1R, dopamine type 1 receptors.

A hypothetical integrative model of the relationships among the main traits of the particular neurobehavioral profiles of the Roman rats is shown in Figure 1. We acknowledge that the proposed model is a simplification and does not take into account the role of other neurotransmitters that may be involved in the neurobehavioral traits that distinguish the Roman lines. It is hypothesized that the lower activity and volume of the mPFC, the HC, and the AMY, together with the higher density of 5-HT2A receptors and the dramatically decreased density of mGlu2 receptors in the frontal cortex of RHA vs. RLA rats may determine a hyper-functional glutamatergic cortical system in RHA rats (Meyza et al., 2009; Klein et al., 2014; Río-Álamos et al., 2017a, 2019; Wood et al., 2017; Tapias-Espinosa et al., 2019). This in turn would elicit an increment in the functional tone of the dopaminergic VTA neurons projecting to the Acb (Elert, 2014) and a decrease in the functional tone of the mesocortical dopaminergic system of RHA rats (Dalley et al., 2007; Giorgi et al., 2007; Probst and van Eimeren, 2013; Tournier et al., 2013; Elert, 2014; Klein et al., 2014; Dalley and Robbins, 2017; Fomsgaard et al., 2018). In addition, the decreased density of DA D2 receptors together with the increased density in DA D1 receptors in the limbic system of RHAs relative to RLA rats (Giorgi et al., 1994, 2007; Guitart-Masip et al., 2006) may also contribute to the more robust functional tone of the mesolimbic dopaminergic system of the former line. It is proposed that this integrated neural circuitry underlies novelty seeking (Tournier et al., 2013), impaired impulse control (Dalley et al., 2007), vulnerability to drug sensitization and abuse (Giorgi et al., 2007; Fattore et al., 2009; Tournier et al., 2013) and schizophrenia-relevant attentional/cognitive impairments (Wakabayashi et al., 2015; Río-Álamos et al., 2019; Tapias-Espinosa et al., 2019; for a review see Swerdlow and Light, 2016). Hence, compared with RLA and heterogeneous/outbred rats, RHAs appear to be unique in that they include all the phenotypes mentioned above, and this makes this rat strain a promising heuristic tool to investigate relationships among these traits and their underlying neurobiological/genetic bases.

Finally, according to the primary addiction hypothesis, the pathophysiologic underpinnings of schizophrenia facilitate the vulnerability to substance use disorder by potentiating the functional activity of the neural circuitry that mediates positive reinforcement thereby leading to the high prevalence of substance use comorbidity in schizophrenics. Hence, the distinct neurobehavioral profile of RHA rats makes this strain a valid model of dual diagnosis schizophrenia.

## Author Contributions

All authors contributed to the bibliographic research, were involved in writing the manuscript, and approved its final version.

## Conflict of Interest Statement

The authors declare that the research was conducted in the absence of any commercial or financial relationships that could be construed as a potential conflict of interest.
